# Evolution of rhizobial siderophore utilization via accessory xeno-siderophore receptors and flexible intake machinery for self-produced siderophores

**DOI:** 10.1093/ismejo/wraf280

**Published:** 2025-12-19

**Authors:** You-Wei Si, Miao-Di Feng, Bo-Sen Yang, Yi-Ning Liu, Ke-Han Liu, Yin Wang, Jian Jiao, Chang-Fu Tian

**Affiliations:** State Key Laboratory of Plant Environmental Resilience, and College of Biological Sciences, China Agricultural University, Beijing, 100193, China; MOA Key Laboratory of Soil Microbiology, and Rhizobium Research Center, China Agricultural University, Beijing, 100193, China; MOE Frontiers Science Center for Molecular Design Breeding, China Agricultural University, Beijing, 100193, China; State Key Laboratory of Plant Environmental Resilience, and College of Biological Sciences, China Agricultural University, Beijing, 100193, China; State Key Laboratory of Plant Environmental Resilience, and College of Biological Sciences, China Agricultural University, Beijing, 100193, China; MOA Key Laboratory of Soil Microbiology, and Rhizobium Research Center, China Agricultural University, Beijing, 100193, China; MOE Frontiers Science Center for Molecular Design Breeding, China Agricultural University, Beijing, 100193, China; State Key Laboratory of Plant Environmental Resilience, and College of Biological Sciences, China Agricultural University, Beijing, 100193, China; State Key Laboratory of Plant Environmental Resilience, and College of Biological Sciences, China Agricultural University, Beijing, 100193, China; MOA Key Laboratory of Soil Microbiology, and Rhizobium Research Center, China Agricultural University, Beijing, 100193, China; State Key Laboratory of Plant Environmental Resilience, and College of Biological Sciences, China Agricultural University, Beijing, 100193, China; State Key Laboratory of Plant Environmental Resilience, and College of Biological Sciences, China Agricultural University, Beijing, 100193, China; MOA Key Laboratory of Soil Microbiology, and Rhizobium Research Center, China Agricultural University, Beijing, 100193, China; Professor Workstation, Anhui Wei Hua Biotechnology Co., Ltd., Suzhou, Anhui, 234000, China; State Key Laboratory of Plant Environmental Resilience, and College of Biological Sciences, China Agricultural University, Beijing, 100193, China; MOA Key Laboratory of Soil Microbiology, and Rhizobium Research Center, China Agricultural University, Beijing, 100193, China; MOE Frontiers Science Center for Molecular Design Breeding, China Agricultural University, Beijing, 100193, China; Professor Workstation, Anhui Wei Hua Biotechnology Co., Ltd., Suzhou, Anhui, 234000, China; Collaborative Innovation Center of Soybean Biotechnology and Nutrition Efficiency, Kaifeng, Henan, 475001, China

**Keywords:** siderophore, exploitation competition, soybean, phylogenomics

## Abstract

*Bradyrhizobium* and *Sinorhizobium* are dominant soybean microsymbionts in acidic/neutral and alkaline soils, respectively. However, the molecular mechanisms underlying this pH-dependent adaptation remain elusive. In this study, phylogenomic analysis of 286 *Bradyrhizobium* and 322 *Sinorhizobium* genomes revealed that *Bradyrhizobium* possesses abundant xeno-siderophore receptors but has limited siderophore biosynthesis functions. In contrast, gene clusters directing siderophore biosynthesis are enriched in *Sinorhizobium*. As siderophores can chelate the prevalent insoluble Fe^3+^ under neutral and alkaline conditions, whereas being less important in acidic environments where soluble Fe^2+^ is readily accessible, we hypothesized that the genus-dependent phyletic distribution of siderophore biosynthesis and exploitation functions may contribute to the pH adaptation of these two genera. Indeed, *Bradyrhizobium* species barely grow under iron-limiting conditions, and this growth defect can be rescued by xeno-siderophores produced by *Sinorhizobium*. Using a xeno-siderophore-exploiting *Bradyrhizobium diazoefficiens* strain, an engineered xeno-siderophore exploiter, and an altruistic siderophore-producing strain derived from *Sinorhizobium fredii*, we revealed the competitive advantage of xeno-siderophore exploitation during soybean nodulation. Heterologous expression of certain *Bradyrhizobium* xeno-siderophore receptors, along with various adaptive mutations in the genome of the *S. fredii* receptor–lacking mutant, allowed this mutant to rapidly restore growth under iron-limiting conditions. These adaptive events in experimental evolution depend on the siderophore biosynthetic function of *S. fredii*. Taken together, these findings suggest that the siderophore utilization ability of soybean rhizobia can be positively selected under iron-limiting conditions: by maintaining abundant xeno-siderophore receptors in acid-tolerant *Bradyrhizobium* and by the rapid adaptive evolution of utilization machinery for self-produced siderophores in alkaline-tolerant *Sinorhizobium*.

## Introduction

Soil microorganisms are crucial for the Earth’s geochemical cycling of multiple elements (e.g. C, N, P, S, and Fe). An increasing number of studies emphasize geographic variations in the distribution and abundance of soil microorganisms with various ecological functions. However, most studies rely on *in situ* observation methods to explore the ecological processes shaping the microbial landscape [1–3]. Within this framework, conclusions regarding geographic patterns are typically derived from correlations between niche characteristics, life-history traits, and genomic information of the studied microorganisms [4–6]. In contrast, the adaptation at the mechanistic molecular level underlying microbial geographic patterns have not been explored as extensively as the *in situ* observational studies, even for model soil microorganisms [3, 7, 8].

Among diverse beneficial soil bacteria, rhizobia stand out due to their high nitrogen fixation ability in association with legumes, making them essential for sustainable agricultural ecosystems [9]. Rhizobia have a saprophytic free-living stage in soils and can induce and infect root nodules on different legumes. Inside nodules, they convert N_2_ into ammonia to support host growth. The first commercial rhizobial inoculant was developed in 1896 and has been widely used in major soybean and forage-producing countries like the USA, Brazil, Argentina, and Australia [10–13]. Globally, common issues affecting rhizobial application efficiency involve competition between inoculants and less-efficient native rhizobial strains [10–15]. There is significant variation in rhizobial biogeographic patterns at the genus, species, and intraspecies levels in the fields of various legume crops [16–18]. *Bradyrhizobium* is characterized by its widespread distribution in soils across six continents worldwide [2] and is a dominant soybean microsymbiont under acidic and neutral conditions, whereas *Sinorhizobium* dominates soybean nodules in alkaline-saline soils in Asia, America, and Europe [19–29]. The interaction between functionally redundant yet taxonomically distinct *Bradyrhizobium* and *Sinorhizobium* can be a crucial factor in shaping their performance as rhizobial inoculants when they encounter each other. Antagonistic competition between *Bradyrhizobium* and *Sinorhizobium* during the nodulation process can be readily postulated, especially under neutral conditions where both can grow well [4, 30]. However, the underlying molecular eco-evolutionary mechanisms have rarely been examined.

This work explored the array of secondary metabolite biosynthesis functions among 286 *Bradyrhizobium* and 322 *Sinorhizobium* genomes. Based on phylogenomic analyses, it was then found that the *Bradyrhizobium* members are short of siderophore biosynthesis functions but enriched with xeno-siderophore receptors of different groups whereas *Sinorhizobium* members are enriched with siderophore biosynthesis gene clusters but harbor few siderophore receptors. Siderophores are low-molecular-weight compounds (500–1500 Da) with high affinity for insoluble ferric iron (Fe^3+^) [31]. Based on the functional moieties that chelate ferric iron, siderophores are categorized into hydroxamates, catecholates, carboxylates, phenolates, and mixed-type variants [32]. In Gram-negative bacteria (including rhizobia), soluble ferric iron siderophore complexes are taken up via outer membrane-integrated specific receptors and ABC transporters (comprising periplasmic and inner membrane components) [31, 33]. Iron plays an essential role in microbial community assembly and host–microbe interactions. Iron–siderophore complexes act as costly public goods that benefit both the producer and other community members, thereby influencing microbial community structure and function by enhancing resource acquisition, inhibiting competitors, and modulating collective behaviors [32]. For example, nonpathogenic corynebacterial species can exploit siderophores produced by the opportunistic pathogen *Staphylococcus aureus*, reducing the abundance of *S. aureus* in the human nasal microbiota [34]. Evidence from closely related marine *Vibrio* strains indicates that xeno-siderophore exploiters (lacking siderophore biosynthesis ability) are more likely to evolve in high-cell-density habitats associated with large particles, whereas low-density free-living *Vibrio* populations harbor more siderophore producers [35]. Closely related siderophore producers exert a more significant inhibitory effect on the plant pathogen *Ralstonia solanacearum*, thereby reducing pathogen prevalence in the rhizosphere [36]. Siderophore exploitation and anti-exploitation shape competition among *Pseudomonas* members in soil and freshwater environments [37]. *Pseudomonas aeruginosa* can enhance its fitness under iron-limiting conditions by evolving to produce alternative siderophores in the presence of isogenic xeno-siderophore exploiters [38]. In interspecies competition with other *Pseudomonas* species, *P. protegens* prefers self-produced siderophores over xeno-siderophores [39]. Reduced environmental availability of public siderophores drives subsequent evolutionary adaptations in iron acquisition strategies and receptor specificity [40, 41].

Among rhizobia, the derepression of siderophore biosynthesis supports nitrogen fixation by *Sinorhizobium meliloti* and *Rhizobium leguminosarum* in nodules of *Medicago* and *Vicia*, respectively [42–46]. By contrast, the soybean microsymbiont model strain *Bradyrhizobium diazoefficiens* USDA110 lacks siderophore biosynthesis capability [40], and the engineered upregulation of siderophore production in *S. fredii* CCBAU45436 impairs rhizobial survival in soybean nodules [33, 47]. Therefore, the global differences in siderophore biosynthesis and receptor-encoding genes between *Bradyrhizobium* and *Sinorhizobium* may not be crucial for nitrogen fixation in soybean nodules. Since Fe^3+^ exists as various insoluble iron oxide compounds in the presence of oxygen and at neutral or alkaline pH [31], we propose a hypothesis encompassing three interconnected aspects as follows: (i) the lack of siderophore biosynthesis capabilities in most *Bradyrhizobium* may reflect their adaptation to acidic conditions, where Fe^2+^ can be readily accessible for uptake and retaining siderophore biosynthesis is not the optimal strategy; (ii) the overrepresentation of siderophore biosynthesis capabilities in *Sinorhizobium* at least partially accounts for their dominance in alkaline conditions, where iron primarily exists as insoluble Fe^3+^; (iii) the enrichment of xeno-siderophore receptors may contribute to *Bradyrhizobium*’s ecological success by effectively exploiting the limited ferric iron–siderophore complexes under neutral conditions, where both acid-tolerant *Bradyrhizobium* and alkaline-tolerant *Sinorhizobium* can survive [19–29, 48]. This hypothesis is supported by the growth phenotypes of representative *Bradyrhizobium* and *Sinorhizobium* strains and their siderophore biosynthetic abilities under different pH conditions. Additionally, this study uncovers the ecological and evolutionary significance of xeno-siderophore exploitation by *Bradyrhizobium*, as well as the rapid adaptive evolution of utilization machineries in *Sinorhizobium* for self-produced siderophores.

## Materials and methods

### Bacterial strains, plasmids, and growth conditions

Rhizobia were cultured in tryptone–yeast extract (TY) medium [49] at 28°C. *Escherichia coli* strains were grown in lysogeny broth (LB) medium at 37°C. A list of the strains employed is summarized in Table S1. The antibiotic concentrations utilized in the study are as follows: nalidixic acid (NA) at 30 μg/ml, trimethoprim (Tmp) at 10 μg/ml, gentamicin (Gen) at 30 μg/ml, kanamycin (Km) at 50 μg/ml, and tetracycline (Tc) at 10 μg/ml. The pH buffering agents chosen for the media are MES (2-morpholinoethanesulphonic acid; pH 5), MOPS (2-morpholinoethanesulphonic acid; pH 7), and AMPSO (N-(1,1-dimethyl-2-hydroxyethyl)-3-amino-2-hydroxypropanesulfonic acid; pH 9), with a concentration of 40 mM. The iron chelator 2,2'-bipyridine was applied at different concentrations (50, 100, 150, and 200 μM) to modulate the concentration of soluble iron. Cell-free supernatant is prepared by centrifuging the culture in the exponential phase (OD_600_ = 1.0) at 6500 rpm and then filtering through a 0.22 μm pore-size filter. The growth curves of the test strains are determined using the Bioscreen C (Oy Growth Curves Ab Ltd, Raisio, Finland).

### Phyletic distribution of biosynthetic gene clusters

The genomes of *Bradyrhizobium* and *Sinorhizobium* strains were obtained from the National Center for Biotechnology Information (NCBI) and processed using anti-SMASH 5.0 [50] with default parameters. The abundance analysis of the various secondary metabolism gene clusters in these two rhizobial genera was carried out and visualized in Ggplot2 [51]. To explore the phyletic distribution of known secondary gene clusters in these rhizobial genera, a hierarchical cluster analysis was conducted using the Pheatmap package in R [52].

### Number and phyletic distribution of siderophore receptors

To identify siderophore receptor homologs in *Sinorhizobium* and *Bradyrhizobium*, the phyletic distribution of experimentally characterized bacterial siderophore receptors was analyzed. A total of 45 experimentally characterized bacterial siderophore receptors were collected from literature as follows: (1) FhuA (NP_414692.1), (2) FhuE (NP_415620.1), (3) FhuE2 (AYK02175.1), (4) FoxA (NP_251156.1), (5) ChtA (PTC33848.1), (6) FiuA (NP_249161.1), (7) FpvB(NP_252857.1), (8) FauA (WP_010926982.1), (9) PutA (AAN56045.2), (10) AvtA (AQV12025.1), (11) BitA (WP_012549028.1), (12) FcuA (CAA47746.1), (13) RhtA (WP_010968208.1), (14) FecA (NP_418711.1), (15) FbsN (WP_049594810.1), (16) FepA (NP_415116.1), (17) Fiu (NP_415326.1), (18) CirA (NP_416660.1), (19) YddB (CBG34449.1), (20) IroN (ADR29866.1), (21) BfhH (ABO12082.2), (22) PiuA (ABO10929.1), (23) PirA (SCX98474.1), (24) BauA (ABO12804.2), (25) PiuD (WP_132667204.1), (26) PfeA (NP_251378.1), (27) FvbA(WP_003093526.1), (28) FrpB (AAF42315.1), (29) IrgA (AAC44766.1), (30) VctA (AAL35618.1), (31) ViuA (AAB86828.1), (32) FprA (WP_014328289.1), (33) FyuA(EFB2704300.1), (34) PupA (AMU01031.1), (35) FptA (NP_252911.1), (36) IutA (WP_000973516.1), (37) FpvA (NP_251088.1), (38) PupB (CAA51995.1), (39) RumA (WNP32538.1), (40) FatA (AAR12527.1), (41) DesE (CAB87217.1), (42) CdtB (CAB76300.1), (43) CchF (QFI40760.1), (44) HtsA (WZL40893.1), and (45) SirA (WP_070051459.1). To avoid confusion regarding the nomenclature of receptors, the protein names reported in the original publications have been used and accession IDs were provided. BLASTP was employed for protein homology comparison of these 45 siderophore receptors against the protein sequences of 56 representative bacterial strains from *Firmicutes, Actinobacteria, Verrucomicrobia*, α-, β-, and γ-*Proteobacteria*. Duplicate and redundant proteins with higher E-values were removed. To illustrate the phyletic distribution of known siderophore receptors, a neighbor-joining phylogenetic tree was constructed using MEGAX [53], based on the RpoB protein sequences of these 56 representative bacterial strains. For *Bradyrhizobium* and *Sinorhizobium* including *B. diazoefficiens* USDA110 (BD110) and *S. fredii* CCBAU45436 (SF45436), the number of known siderophore receptors for different siderophore categories per genome was further analyzed (with identity values above 30%, *E*-values below 10^−5^, and query coverage above 40%).

### Quantitative detection of siderophore production

Siderophore production was determined by employing a universal chemical assay with Chrome Azurol S (CAS). The detailed procedures are as follows: the strains were cultivated in TY medium at various pH levels. Next, the CAS detection solution was added in a 1:1 ratio to the sterile cell-free supernatant and allowed to react for two hours. The optical density (OD) at 630 nm was measured using a microplate reader (Spark; Tecan, Switzerland), yielding the value *A*. For the control, the corresponding sterile medium without microbial cultivation at the same pH was utilized and mixed with the CAS detection solution in a 1:1 ratio. It was also allowed to react for 2 h, and the OD_630_ was measured, resulting in the value *Ar*. The formula for calculating the relative content of siderophores (SU%) is as follows: SU% = (1 − *A/Ar*) × 100% [54].

### Genetic procedures

Seamless cloning was employed to construct various plasmids for genetic manipulation. The upstream and downstream fragments of the target gene were amplified using high-fidelity Polymerase Chain Reaction (PCR) enzymes with the pairs of primers listed in Table S2. Subsequently, these fragments were ligated to the linearized pJQ200SK [55] using a seamless cloning kit (Taihe Biotechnology, Beijing, China). The pJQ200SK-derived plasmids harbored by positive clones were verified by PCR and Sanger sequencing and introduced into rhizobia with the help of the helper plasmid pRK2013 [56]. Single-crossover clones were obtained by gentamicin resistance screening and subsequently subcultured on TY agar plates with 5% sucrose for counter-selection of double recombinants. To generate Green Fluorescent Protein (GFP) -tagged BD110 and SF45436 strains, the pJQ200SK-derived plasmid carrying the Paph-GFP sequence amplified from pRJPaph-bjGFP [57] and up-/downstream fragments was constructed. Downstream procedures followed those in the gene deletion experiment. To construct strains for heterologous expression of the BD110 siderophore receptor gene (SR1−8), fragments containing the corresponding coding sequence and the promoter region of *fprA* were cloned into the pBBR1MCS-5 vector [58]. The pBBR1MCS-5 derivative vectors with correct sequences were introduced into rhizobia by conjugation. Point mutations were designed at the 5' end of the primers listed in Table S2, and up-/downstream fragments with point mutations were synthesized by high-fidelity PCR enzymes and then ligated into the pJQ200SK via homologous recombination. Resultant pJQ200SK derivatives were introduced into rhizobia to obtain strains carrying desired point mutations. All in-frame deletion mutants, tagged strains, heterologous expression strains, and those with point mutations were verified by colony PCR and Sanger sequencing.

### Competitive nodulation

Cultivated soybean (*Glycine max* cv. JD17) seeds were treated with 95% ethanol for 30–60 s. Subsequently, surface sterilization was performed with 20% (w/v) sodium hypochlorite for 2–3 min. After that, the seeds were washed six to eight times with autoclaved deionized water and germinated on 0.8% agar plates at 28°C in the dark for 48 h. Overnight rhizobial cultures were adjusted to an OD_600_ of 0.2 in 0.85% NaCl solution. To assess the nodule occupancy of rhizobia, BD110-GFP and SF45436Δ*asbA*-GFP were each individually mixed with SF45436 or SF45436Δ*fprAfoxA* in ratios of 1:1 (OD_600_ = 0.2). These mixed cultures were inoculated onto soybean plants grown in vermiculite (within Leonard jars) moistened with a low-nitrogen nutrient solution [per 1 l medium: Ca(NO_3_)_2_·4H_2_O 0.03 g, KCl 0.075 g, MgSO_4_·7H_2_O 0.06 g, K_2_HPO_4_ 0.136 g, CaSO_4_·2H_2_O 0.46 g, H_3_BO_3_ 2.86 mg, MnSO_4_ 1.81 mg, CuSO_4_·5H_2_O 0.8 mg, ZnSO_4_ 0.22 mg, H_2_MoO_4_ 0.02 mg, and ethylenediaminetetraacetic acid (EDTA) -Fe^3+^ 0.11 g in 1 l medium] at pH 7. The low-nitrogen nutrient solution contained 300 μM EDTA-Fe^3+^. To assess nodulation occupancy under iron-limiting conditions, a modified low-nitrogen nutrient solution (omitting EDTA-Fe^3+^) was also used. The plants were grown at 24°C under a 12-h light/12-h dark cycle. At 30 dpi, the nodule occupancy of rhizobia was evaluated by GFP fluorescence detection.

### RNA extraction and Reverse Transcription quantitative Polymerase Chain Reaction (RT-qPCR)

**Figure 1:**
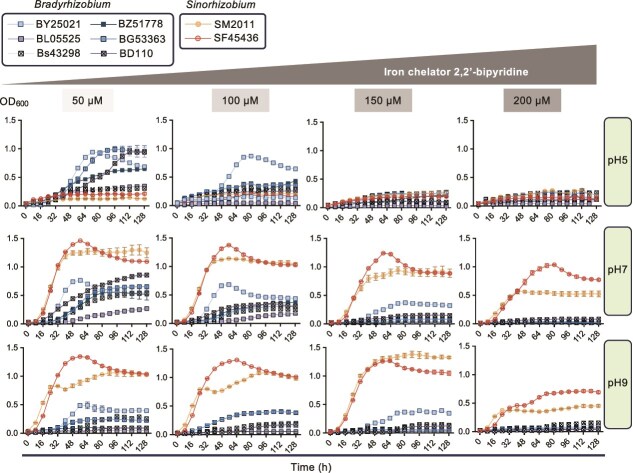
Growth curves of *Bradyrhizobium* and *Sinorhizobium* representatives under different pH and iron-limiting conditions. SM, *Sinorhizobium meliloti*; SF, *Sinorhizobium fredii*; BY, *Bradyrhizobium yuanmingense*; BL, *Bradyrhizobium liaoningense*; Bs, *Bradyrhizobium* sp. BZ, *Bradyrhizobium zhanjiangense*; BG, *Bradyrhizobium guangxiense*; BD, *Bradyrhizobium diazoefficiens*. Error bars represent SD of three biological replicates (not visible for those small SD values).

Among the tested concentrations of 2,2'-bipyridine (50, 100, 150, and 200 μM), it was found that 150 μM 2,2'-bipyridine was sufficient to induce iron-limiting effects without killing siderophore biosynthesis-deficient rhizobia at pH 7 and pH 9 (Fig. 1). In order to test the transcriptional level of transferred siderophore receptor genes in SF45436 backgrounds, rhizobial cultures were inoculated into TY medium containing 150 μM 2,2'-bipyridyl, and cells were collected at OD_600_ of 0.8. Total RNA was extracted from the cells using the Bacteria Total RNAKit (Zomanbio). Genomic DNA was removed, and complementary DNA (cDNA) was synthesized by reverse transcription using the FastKing RT Kit with gDNase (Tiangen). RT-qPCR was performed using SYBR Green Real-time PCR Mix (GenStar) and an ABI QuantStudio 6 Flex System. The 16S ribosomal RNA (rRNA) gene was used as an internal control to normalize the relative transcription levels of target genes. Primers used for RT-qPCR are listed in Table S2.

### Experimental evolution and genome resequencing

Experimental evolution was used to investigate whether a xeno-siderophore receptor from BD110 could enhance siderophore acquisition in SF45436Δ*fprA* (which lacks the canonical receptor FprA for its own siderophore petrobactin) under iron-limiting conditions and to identify potential adaptive mutations arising under these selective pressures. The wild-type SF45436, SF45436Δ*fprA*, and its derivatives harboring either the empty vector pBBR1-MCS5 (pB) or those carrying individual siderophore receptor genes (SR1–8) from BD110 were cultured. The evolutionary experiment was performed using a fully automated microbial growth curve analyzer (the Bioscreen C system). Cultures were propagated in iron-limiting TY medium containing 150 μM 2,2'-bipyridine at 28°C with shaking at the instrument’s medium speed for 7 days. The cultures collected at 128 h postinoculation were plated on the solid TY medium, and three independent colonies were reinoculated into the iron-limiting TY medium to verify the growth phenotype. Genomes of the evolved clones were resequenced on an Illumina NovaSeq platform with paired-end reads (Shanghai Personalbio Technology Co., Ltd.).

High-quality reads were aligned to the reference genome using the Burrows-Wheeler Alignment (BWA) program with default parameters [59]. The resulting SAM files were sorted and converted to Sequence Alignment/Map (BAM) files using Picard [59], ensuring consistency of paired-end read information with the “FixMateInformation” command and removing duplicates with the “MarkDuplicates” command. Single-nucleotide polymorphism (SNP) detection was performed using the GATK software [60], followed by realignment of reads around all InDels (Insertions and Deletions) with the “IndelRealigner” command to improve SNP prediction accuracy. Copy number variations were detected using CNVnator [61] with a bin_size of 100 bp. Results were filtered to remove multi-copy regions and retain regions with Read Depth (RD) values ≤0.05 or ≥1.8 (normal chromosomal regions defined as RD = 1). Finally, chromosomal structural variations were detected using BreakDancer [62] based on the paired-end mapping principle.

### Sequence analyses of adaptive mutations

Secondary and 3D structures of proteins encoded by the mutated and wild-type genes were predicted by JPred4 [63] and AlphaFold 3.0 [64], respectively. Subcellular localization was predicted using PSORTb v3.0 [65], CELLO v.2.5 [66], and DeepLoc 2.0 [67]. Docking simulations were performed using Schrodinger Maestro 11.5 (Schrödinger Release 2025-4: BioLuminate, Schrödinger, LLC, New York, NY, 2025) or AutoDock Vina method via SwissDock as indicated in related figure legends [68].

## Results and discussion

### Genus-dependent phyletic distribution of siderophore biosynthesis functions in *Sinorhizobium* and *Bradyrhizobium*

The antiSMASH database was employed to predict biosynthetic gene clusters potentially directing the biosynthesis of secondary metabolites among 286 *Bradyrhizobium* and 322 *Sinorhizobium* genomes accessible in the public database. The top five most abundant biosynthetic gene clusters identified either from 278 *Bradyrhizobium* or 210 *Sinorhizobium* genomes were used for hierarchical cluster analysis (Fig. S1A). There are 234 and 14 *Bradyrhizobium* genomes harboring gene clusters belonging to NRPSs (nonribosomal peptide synthetases) siderophores and those involved in the biosynthesis of NRPS-independent (NIS) siderophores, respectively. In contrast, there are 20 and 156 *Sinorhizobium* genomes containing gene clusters related to NRPS- and NIS-siderophores, respectively. NRPSs are large multi-enzyme complexes that catalyze biosynthesis in three steps: initiation, condensation, and termination [69]. The NRPS-siderophore-related genes abundant in *Bradyrhizobium* are involved in the biosynthesis of siderophore malleobactin, which is widely distributed in *Burkholderia* of β*-Proteobacteria* class [70]. Specifically, homologs of NRPS MbaAB with activities including adenylation, thiolation, condensation, epimerase, and thioesterase are detected in 85 out of 278 *Bradyrhizobium* genomes. Among these *mbaAB*-harboring strains, 41 also have *mbaC* (ornithine monooxygenase) and *mbaH* (hydroxylase) that are involved in the modification of the backbone. However, *mbaE* (formyltransferase), which is essential for obtaining the major malleobactin A and its hydroxylamine (B), nitroso (C), and azoxy-linked dimer (D) derivatives [70], is absent in all *Bradyrhizobium* genomes (Fig. S1B). Therefore, those *Bradyrhizobium* strains harboring only *mbaAB* (85 strains) or *mbaABCH* (41 strains) may not produce the functionally characterized malleobactins A–D. NIS siderophore gene clusters identified in *Sinorhizobium* include the complete petrobactin [33, 71] and rhizobactin [72] gene clusters functionally characterized in *S. fredii* and *S. meliloti*, respectively.

These findings suggest that a majority of *Bradyrhizobium* strains may not produce siderophores, whereas a considerable number of *Sinorhizobium* species may benefit from their complete NIS siderophore biosynthesis and utilization pathways. To test this hypothesis, the growth curves and siderophore production of representative *Bradyrhizobium* and *Sinorhizobium* strains were measured under different pH (5, 7, and 9) and iron-limiting conditions. Five of the six tested *Bradyrhizobium* species could grow at pH 5 in the rich TY medium, and their growth was significantly reduced when the concentration of the iron-chelator 2,2'-dipyridyl was increased from 50 to 100 to 150 μM (Fig. 1). *Bradyrhizobium yuanmingense* CCBAU25021 (BY25021) grew more vigorously than the other *Bradyrhizobium* strains at pH 5 under iron-limiting conditions (Fig. 1; 100 μM 2,2'-dipyridyl). At pH 7, where all tested *Bradyrhizobium* strains can grow, BY25021 displayed a superior growth phenotype compared to other *Bradyrhizobium* strains under iron-limiting conditions (100 and 150 μM 2,2'-dipyridyl; Fig. 1). At pH 9, all tested *Bradyrhizobium* strains exhibited a weak growth phenotype (Fig. 1). Among the tested *Bradyrhizobium* strains, only BY25021 harbors a putative dimethylcoprogen siderophore biosynthetic gene cluster [73]. BY25021 was among 41 out of 278 *Bradyrhizobium* strains harboring *mbaABCH* genes, whereas the other five test strains contained fewer *mba* genes encoding key proteins in malleobactin synthesis and modification: none (BG53363), *mbaBC* (Bs43298), *mbaAB* (BL05525), or *mbaABC* (BZ51778 and BD110). Independently, *Paraburkholderia phymatum* STM815 also carries an *mba* gene cluster lacking *mbaE* (encoding formyltransferase) but possessing additional *phmK* and *phmL* (encoding acetyltransferases), which are involved in producing the malleobactin-like siderophore phymabactin [74–76].

**Figure 2:**
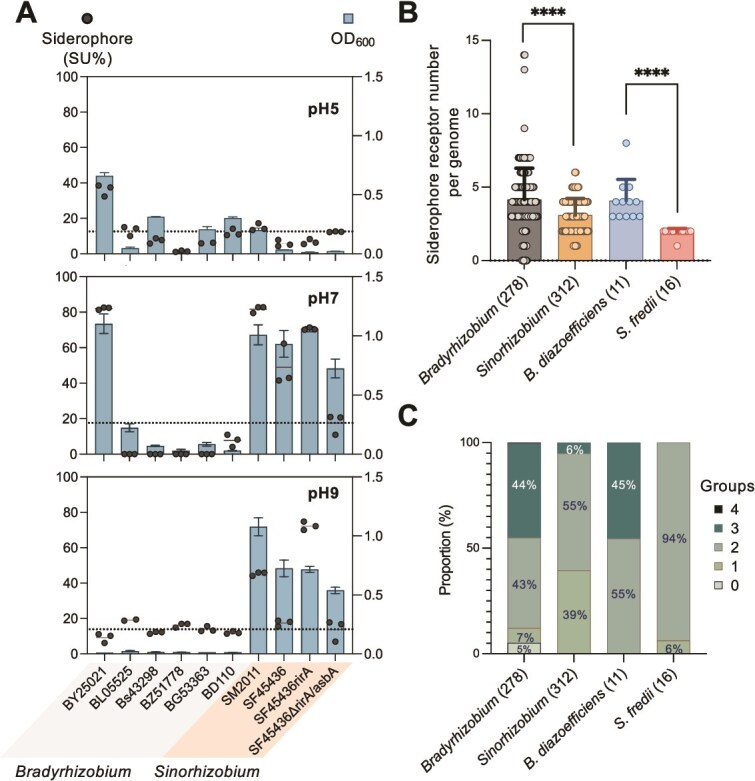
*Bradyrhizobium* rarely produce siderophores under iron-limiting conditions but harbor more siderophore receptors. (A) Siderophore production assay under different pH conditions. 150 μM iron chelator 2,2'-bipyridine was added to pH 7 and pH 9 TY medium whereas 100 μM 2,2'-bipyridine was added to pH 5 TY medium. No rhizobia was able to grow in pH 5 medium with 150 μM bipyridine. Error bars represent SD of three biological replicates (not visible for those small SD values). SM, *Sinorhizobium meliloti*; SF, *Sinorhizobium fredii*; BY, *Bradyrhizobium yuanmingense*; BL, *Bradyrhizobium liaoningense*; Bs, *Bradyrhizobium* sp. BZ, *Bradyrhizobium zhanjiangense*; BG, *Bradyrhizobium guangxiense*; BD, *Bradyrhizobium diazoefficiens*. The *rirA* mutant and *∆rirA/asbA* double mutant derived from *S. fredii* SF45436 were used as siderophore overproducing and deficient controls. The dashed line represents the average values of the siderophore-nonproducing strain SF45436 *∆rirA/asbA*. (B) The total number of receptors per genome. The number of analyzed genomes (in brackets) and significant difference between means are indicated (Kolmogorov–Smirnov test, ****, *P* < .0001). (C) The proportion of genomes with indicated number of receptor groups corresponding to siderophore categories (hydroxamates, catecholates, carboxylates, phenolates, and mixed-type variants) per genome.

*Sinorhizobium meliloti* and *S. fredii* strains can grow well at pH 7 and pH 9, and increasing concentrations of iron-chelator from 50 to 100 to 150 to 200 μM had a less significant impact on their growth compared to *Bradyrhizobium* strains (Fig. 1). These growth curves are consistent with the siderophore production abilities of test strains, particularly at pH 7 where both *Bradyrhizobium* and *Sinorhizobium* can grow well (Figs 1 and 2). A considerable level of extracellular siderophores was detected in representative strains of *S. meliloti* (SM2011) and *S. fredii* (SF45436), which have complete biosynthesis pathways for rhizobactin [72] and petrobactin [33], respectively.

Siderophores were not detected from cultures of *Bradyrhizobium* strains except BY25021 (Fig. 2). The low incidence of siderophore production in *Bradyrhizobium* strains is also confirmed by an earlier study in which only 1 out of 20 tested *Bradyrhizobium* strains produces siderophores under iron-limiting conditions [77]. BY25021 was isolated from nodules of wild soybean grown in alkaline soils, and this species (*B. yuanmingense*) is more likely to inhabit nodules of wild and cultivated soybeans in alkaline soils than *B. liaoningense, B. japonicum, B. diazoefficiens*, and *B. elkanii* [30, 78]. In contrast, *S. fredii* is the dominant microsymbionts of wild and cultivated soybeans in alkaline-saline soils [30, 78]. These biogeographic patterns and growth phenotypes are in line with the fact that the solubility of iron decreases at neutral pH conditions, forming insoluble iron oxide compounds [48]. Under these iron-limiting conditions, various siderophores produced by microorganisms can form soluble Fe^3+^ complexes that are then actively taken up via siderophore receptors to meet their cellular requirements [31]. However, it remains unclear why most *Bradyrhizobium* members lacking siderophore biosynthesis functions can dominate in the iron-limiting neutral soils [2, 16, 30, 78].

### *Bradyrhizobium* possessing more siderophore receptors exploits the siderophores produced by *Sinorhizobium* in free-living circumstances

Although, as demonstrated above, siderophore biosynthesis functions are just occasionally found in *Bradyrhizobium*, species of this genus dominate in the rhizosphere and nodules of soybeans grown in iron-limiting neutral pH soils [4, 16]. This prompts us to test whether *Bradyrhizobium* acts as an exploiter for xeno-siderophores produced by co-existing siderophore producers under iron-limiting neutral conditions [32, 69]. To identify siderophore receptor homologs in *Bradyrhizobium*, 45 experimentally characterized siderophore receptors from diverse bacteria were analyzed (Fig. S2). These documented siderophore receptors were assigned to different groups corresponding to the major siderophore categories, which these receptors have been shown to recognize in the literature (Fig. S2; hydroxamates, catecholates, carboxylates, phenolates, and mixed-type variants). On average, *Bradyrhizobium* and *Sinorhizobium* have 4.16 and 3.10 siderophore receptor homologs per genome, respectively (*P* < .0001; Fig. 2B). Additionally, 44% *Bradyrhizobium* strains and 6% *Sinorhizobium* strains have three groups of siderophore receptor homologs per genome (Fig. 2C). *Sinorhizobium fredii*, which is the major soybean microsymbiont in alkaline–saline soils [30, 78], harbors 1.91 siderophore receptor homologs of one to two groups per genome (Fig. 2B and C). By contrast, the widely distributed soybean rhizobia *B. diazoefficiens* has 4.09 receptor homologs of two to three groups (Fig. 2B and C). There are eight putative siderophore receptors in the well-known soybean inoculant, *B. diazoefficiens* USDA110 (BD110) [79]. These BD110 siderophore receptors (SRs) include four hydroxamate receptors, FegA (SR6; FiuA homolog) [80, 81], FhuE (SR7; FpvB homolog) [40, 81], and FiuA homologs WP_038967578.1 (SR3) and WP_011090691.1 (SR8) [81], and four catecholate receptors, EntR (SR2; PiuD homolog) [40], PirA homolog (SR4) [82], Fiu homolog (SR1) [83], and FepA homolog (SR5) [84].

**Figure 3:**
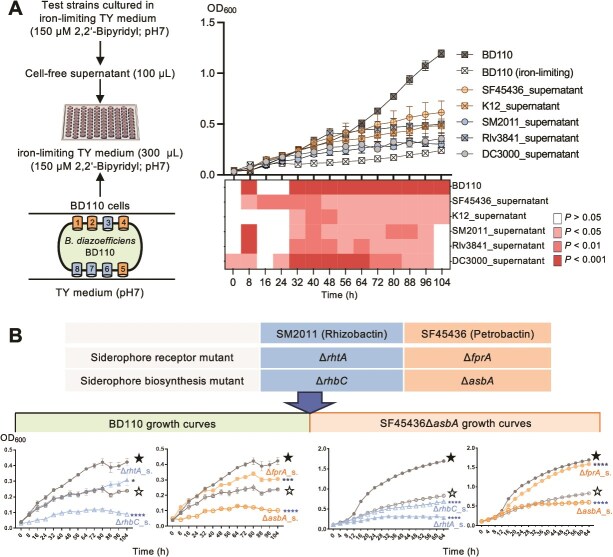
Cell-free supernatant of known siderophore producers promotes *B. diazoefficiens* BD110 growth under iron-limiting conditions. (A) Growth curves of BD110 with or without cell-free supernatant from known siderophore producers. Eight putative siderophore receptors for catecholate (1, 2, 4, and 5) or hydroxamate (3, 6, 7, and 8) siderophores are indicated in BD110 cells. Error bars represent SD of three biological replicates (not visible for those small SD values). Positive control: growth of BD110 in TY medium; negative control: growth of BD110 in the iron-limiting TY medium. Heatmap shows *P*-values based on two-way ANOVA followed by Dunnett’s multiple comparisons test with BD110 (iron-limiting) as the control. (B) Fitness enhancement of BD110 and SF45436Δ*asbA* by cell-free supernatant of siderophore producers depends on producers’ siderophore biosynthetic genes. Error bars represent the Standard Error of the Mean (SEM) of three biological replicates (not visible for those small SEM values). ★, positive control: growth curve of BD110 or SF45436Δ*asbA* in TY medium; ☆, negative control: growth curve of BD110 or SF45436Δ*asbA* in the iron-limiting TY medium. Solid symbols indicate the growth curve of BD110 or SF45436Δ*asbA* with cell-free supernatant from siderophore-producing but nonabsorbing mutants (Δ*rhtA_*s. and Δ*fprA_*s.); hollow symbols indicate the growth curve of BD110 and SF45436Δ*asbA* with cell-free supernatant from mutants that don’t produce siderophores (Δ*rhbC_*s. and Δ*asbA*_s.) (two-way ANOVA followed by Sidak’s multiple comparisons test, alpha = 0.05).

It has been reported that *R. leguminosarum* 3841 (RL3841), *S. meliloti* 2011 (SM2011), *S. fredii* CCBAU45436 (SF45436), *E. coli* K12 (K12), and *Pseudomonas syringae* DC3000 (DC3000) synthesize vicibactin (hydroxamate) [85], rhizobactin (hydroxamate) [72], petrobactin (catecholate) [33, 71], enterobactin (catecholate) [86], and pyoverdine (a catechol and two hydroxamate-chelating sites) [87], respectively (Fig. S3). To test the potential xeno-siderophore utilization abilities of BD110, the cell-free supernatant of RL3841, SM2011, SF45436, K12, and DC3000 grown under iron-limiting conditions (pH 7) was combined with BD110 cells. The resulting mixture was then further grown in the iron-limiting TY medium (Fig. 3A). It was found that the cell-free supernatant of these test strains could significantly enhance the growth of BD110 in the iron-limiting TY medium to different degrees [Fig. 3A; two-way Analysis of Variance (ANOVA), *P*-values <.0001]. Higher cell density than the negative control (BD110 cells in the iron-limiting medium) was observed at 104 h postcultivation for all treatments (Fig. 3A; Sidak’s multiple comparisons test, *P*-values <.01), except for the SM2011_supernatant treatment.

The supernatant contains a cocktail of diverse metabolites, and the growth improvement of BD111 under iron-limiting conditions can reflect a combined effect. To further assess whether the beneficial effect of cell-free supernatants from siderophore producers depends partially on siderophore biosynthesis, in-frame deletion mutants Δ*rhbC* and Δ*asbA* were constructed in SM2011 and SF45436, respectively. These two genes are essential for siderophore biosynthesis by these two strains [33, 72]. The growth of BD110 in the presence of cell-free supernatant from both SM2011Δ*rhbC* and SF45436Δ*asbA* mutants was worse than that of the negative control (without supernatant from cultures of other strains) (Fig. 3B). This might be because soluble iron in the supernatant has been heavily utilized by these siderophore nonproducing mutants. The mutations in siderophore biosynthesis genes do not impact siderophore uptake [33, 72]. Consequently, related mutants such as SM2011Δ*rhbC* and SF45436Δ*asbA* can be regarded as xeno-siderophore exploiters like BD110. By contrast, mutants of siderophore receptors can function as altruistic members if they can produce public siderophores in a community [32]. Here, SM2011Δ*rhtA* and SF45436Δ*fprA* were constructed as such altruistic mutants lacking their rhizobactin and petrobactin receptors but producing rhizobactin and petrobactin, respectively [33, 72]. The cell-free supernatant from these two mutants grown in the iron-limiting TY medium promoted the growth of BD110 under the same conditions (Fig. 3B; two-way ANOVA and Sidak’s multiple comparisons test, *P*-values <.05). Moreover, SF45436Δ*fprA* functioned as a superior altruistic mutant relative to SM2011Δ*rhtA* for the growth of the xeno-siderophore exploiter BD110 (Fig. 3B). When BD110 was replaced by the engineered xeno-siderophore exploiter SF45436Δ*asbA*, the supernatant from SF45436Δ*fprA* rather than that from SM2011Δ*rhtA* had a significant positive effect on the growth of SF45436Δ*asbA* in the iron-limiting TY medium (Fig. 3B; two-way ANOVA and Sidak’s multiple comparisons test, *P*-values <.0001). These results imply that the widely used soybean inoculant strain BD110 can exploit xeno-siderophores more efficiently from its competitor SF45436 than from the *Medicago* microsymbiont SM2011. Additionally, SF45436 failed to gain any benefit from the specific xeno-siderophore secreted by SM2011. Thus, given the knowledge of the specificity between receptors and siderophore structures [31], these findings support that more siderophore receptors of different groups likely provide BD110 with an ecological advantage over SF45436 under iron-limiting neutral conditions.

### Soybean rhizobia gain advantages by exploiting xeno-siderophores during nodulation

Surviving in bulk soil and the rhizosphere of legumes does not ensure the success of rhizobial inoculants. There is intense antagonistic competition between different rhizobial strains during the stage of nodulation [88, 89]. In three independent studies on nodule occupancy of soybean, alfalfa, and *Lotus japonicum* [90–92], most legume nodules are singly infected by a single rhizobial clone, which supports the strong bottleneck effect of the nodulation process. Independent common garden experiments in Argentina, Brazil, China, and Spain have shown that *Bradyrhizobium* strains outcompete *Sinorhizobium* in competitive nodulation on soybeans under acidic and neutral conditions. In contrast, *Sinorhizobium* is more competitive under alkaline conditions [29, 93–96]. However, the underlying mechanisms remain unclear.

**Figure 4:**
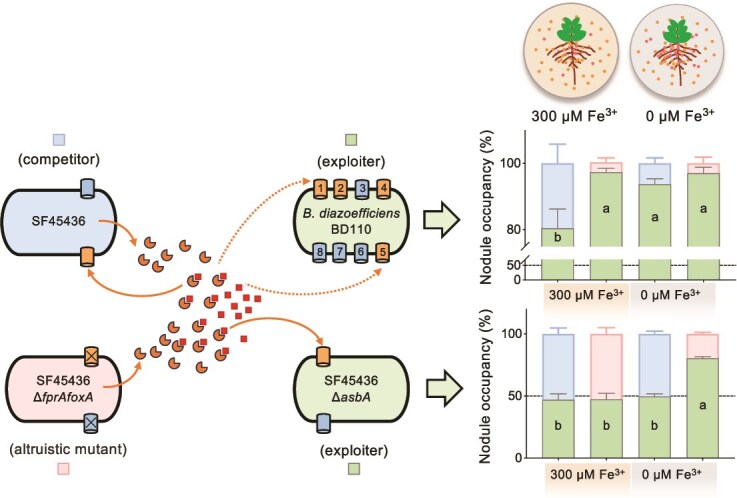
Natural and engineered xeno-siderophore exploiters show higher nodule occupancy than competitors. Error bars represent the SD of nine biological replicates. Different letters indicate a significant difference based on ANOVA followed by Tukey’s multiple comparisons test (alpha = 0.05). The growth medium was supplied with 300 or 0 μM Fe^3+^.

Iron is a limited resource in circumneutral pH soils, and both plants and soil microorganisms compete for available iron [97, 98]. We wondered whether the siderophore exploitation ability contributes to rhizobial competitive nodulation on soybean plants in the presence of a siderophore producer under neutral pH conditions. For this purpose, the xeno-siderophore exploiter BD110-GFP was mixed with SF45436 or SF45436Δ*fprAfoxA* in a 1:1 ratio in the inoculant (Fig. 4). FoxA is another characterized *Sinorhizobium* receptor for xeno-siderophore (hydroxamate) [99]. SF45436Δ*fprAfoxA*, lacking two siderophore receptors, can be regarded as more altruistic than the siderophore competitor SF45436 that produces and imports self-produced petrobactin to exploit environmental iron [33]. Indeed, in the presence of a standard low-nitrogen nutrient solution containing 300 μM Fe^3^, the nodule occupancy of BD110-GFP was higher when co-inoculated with the altruistic mutant SF45436Δ*fprAfoxA* than when co-inoculated with SF45436. This indicates that siderophore utilization was functional in both SF45436 and BD110-GFP, and this soybean growth condition may represent an iron-limiting environment to a certain extent. However, the difference in the effects of SF45436 and SF45436Δ*fprAfoxA* on the nodule occupancy of BD110 was not statistically significant when Fe^3+^ was omitted from the low-nitrogen nutrient solution. This is because BD110-GFP showed a higher nodulation occupancy when co-inoculated with the siderophore competitor SF45436 under Fe^3+^-depleted conditions than in the presence of 300 μM Fe^3+^ (ANOVA followed by Tukey’s multiple comparisons test, *P*-value <.05). Given the considerable genomic difference between strains BD110 and SF45436, BD110-GFP was subsequently replaced by the engineered siderophore exploiter SF45436Δ*asbA*-GFP. The nodule occupancy of SF45436Δ*asbA*-GFP was higher under Fe^3+^-depleted conditions when co-inoculated with the altruistic mutant SF45436Δ*fprAfoxA* but not when co-inoculated with the parental strain SF45436 (ANOVA followed by Tukey’s multiple comparisons test, *P*-value <.05). As strains expressing GFP may be at a disadvantage due to unnecessary metabolic burden, the observed high nodule occupancy of BD110-GFP and SF45436Δ*asbA*-GFP may be slightly underestimated. The notable higher nodule occupancy of the natural BD110-GFP than SF45436Δ*asbA*-GFP in all test treatments (Fig. 4) may be at least partially explained by their arsenal of siderophore receptors. BD110-GFP has eight xeno-siderophore receptors, whereas SF45436Δ*asbA*-GFP has one xeno-siderophore receptor and one receptor for self-produced petrobactin. Additionally, *Bradyrhizobium* members usually have a larger genome size and a longer generation time than *Sinorhizobium* [4, 100, 101], and the slow-growing trait can be an advantage when nutrients are limited or in the presence of other stresses [102].

### Rapid experimental evolution of the machinery utilizing self-produced siderophores

**Figure 5:**
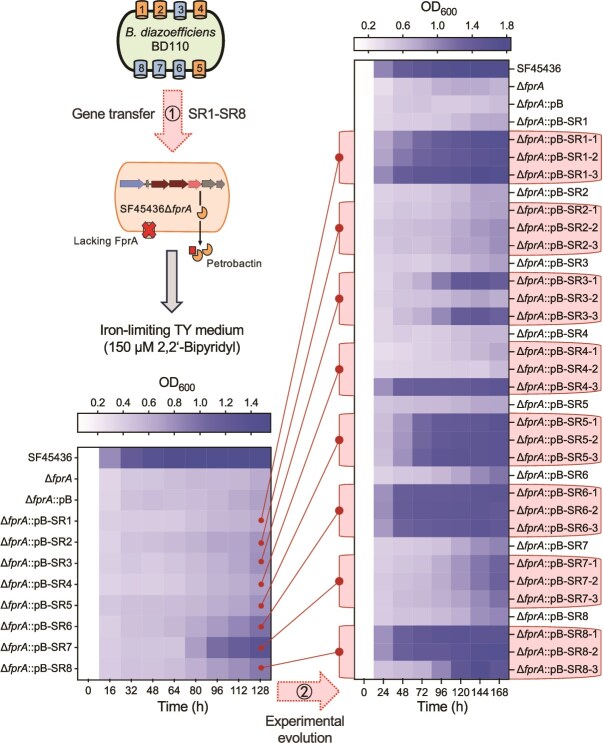
Growth phenotypes of SF45436Δ*fprA* derivatives carrying BD110 siderophore receptor genes under iron-limiting conditions. ①, the plasmid pBBR1MCS-5 (pB) carrying individual siderophore receptor genes (SR1–SR8) was transferred into SF45436Δ*fprA*; the resultant strains were grown under iron-limiting conditions (nine biological replicates). Three independent clones were collected at 128 h postcultivation and further cultured under the same conditions (four biological replicates) in the experimental evolution scenario (②). The OD_600_ values were based on the mean of all biological replicates. Siderophore receptors (SR1-SR8) from BD110 include four hydroxamate receptors FegA (SR6; FiuA homolog), FhuE (SR7; FpvB homolog), and FiuA homologs WP_038967578.1 (SR3) and WP_011090691.1 (SR8), and four catecholate receptors EntR (SR2; PiuD homolog), PirA homolog (SR4), Fiu homolog (SR1), and FepA homolog (SR5). Gene IDs for SR1-SR8 are *blr3555, blr3904, blr4504, bll4766, bll4881, bll4920, bll6183*, and *bll7968*. Evolved strains are labeled with “-1,” “-2,” and “-3.”

**Figure 6:**
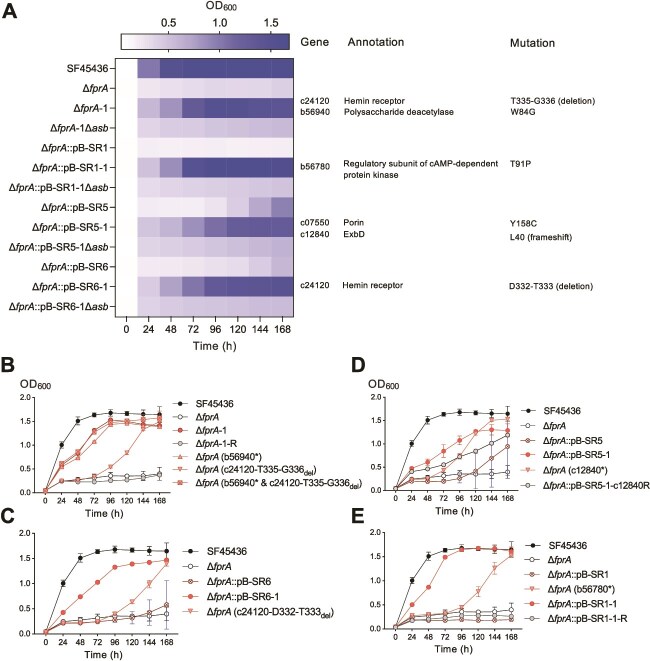
Fast adaptive evolution of SF45436Δ*fprA* derivatives under iron-limiting conditions depends on the siderophore biosynthesis function and adaptive mutations. (A) Growth phenotype of evolved clones and their derivatives lacking the siderophore biosynthesis gene *asbA*. Various mutations identified in evolved clones were determined by genome resequencing. (B–E) Genetic verification of adaptive mutations identified in representative evolved clones. The OD_600_ values were based on the mean of three biological replicates for all test strains, and SD is shown in (B–E). Data used in (A) and (B–E) were obtained in the same experiment. Large variation observed at later sampling time points of Δ*fprA*::pB-SR5 and Δ*fprA*::pB-SR6, indicating potential newly evolved clones in certain biological replicates (C, D). Evolved strains are labeled with “-1.” “-R” indicates the complemented strain. * and “del” indicate adaptive mutations identified in the evolved strains.

The results on BD110 and SF45436 provide strong evidence that naturally evolved xeno-siderophore exploitation ability is crucial for rhizobial survival and competitive nodulation under iron-limiting neutral conditions in the presence of siderophore-producing strains. We therefore wondered whether siderophore receptors from the natural xeno-siderophore exploiter BD110 could replace FprA, the canonical petrobactin receptor of SF45436, to allow SF45436 to thrive under iron-limiting conditions. To this end, genes encoding eight siderophore receptors from BD110 (SR1–8) were individually cloned and transferred into SF45436Δ*fprA* (Fig. 5) that lacks the specific receptor FprA for the siderophore petrobactin [33]. In the iron-limiting TY medium (150 μM 2,2'-bipyridyl), transcription products of all transferred siderophore receptor genes SR1–8 were detected in SF45436Δ*fprA* derivatives, though SR2 had a relatively low expression level (Fig. S4). Among nine biological replicates of each SF45436Δ*fprA* derivative carrying individual siderophore receptor genes (SR1–SR8), slight enhancement of late-stage growth was occasionally observed compared to SF45436Δ*fprA* (Fig. 5). This suggests potential adaptive mutations arising in these SF45436Δ*fprA* derivatives under selective pressures. To enrich potential adaptive clones, cultures collected at 128 h postinoculation were plated on the solid TY medium and three independent clones of each test strain were collected and re-cultured in the iron-limiting TY medium. Growth phenotype like the wild-type SF45436 was observed for certain evolved clones of SF45436Δ*fprA* derivatives carrying SR1, SR3, SR4, SR5, SR6, and SR8 (Fig. 5). Independent experiments also identified evolved clones of SF45436Δ*fprA* and SF45436Δ*fprA* carrying the empty vector that showed enhanced growth phenotype under iron-limiting conditions (Fig. S5). Among these evolved clones, representatives with reproducible and robust growth phenotypes in independent experiments under iron-limiting conditions were subject to genome sequencing (Fig. 6). Nonsynonymous substitution, deletion, and frameshift mutations were identified in genes encoding a regulatory subunit of cyclic Adenosine Monophosphate (cAMP) -dependent protein kinase (b56780), polysaccharide deacetylase (b56940), porin (c07550), ExbD (c12840), and a putative receptor for hemin (c24120). To test whether these evolved clones (with enhanced growth under iron-limiting conditions) depend on the self-produced siderophore petrobactin, the petrobactin biosynthesis gene *asbA* was further deleted in these clones. Indeed, the resulting *asbA*-deficient derivatives of these evolved clones exhibited a notable reduction in growth under iron-limiting conditions (Fig. 6A). Thus, this indicates that the rapid adaptive evolution of SF45436Δ*fprA* derivatives under the tested iron-limiting conditions depends on petrobactin biosynthesis. These adaptive mutations are involved in the utilization of the self-produced siderophore petrobactin.

Reverse genetics analysis showed that the in-frame deletion of T335-G336 (identified in SF45436Δ*fprA*-1) or D332-T333 in c24120 (identified in SF45436Δ*fprA*::pB-SR6-1) can partially enhance the growth of SF45436Δ*fprA* (Fig. 6B and C). c24120 is a putative receptor for hemin and is under direct regulation of iron-responsive regulator RirA [33]. Both T335-G336 and D332-T333 in-frame deletions are within the β-barrel domain (Fig. S6) that is inserted into the outer membrane and functions as a conserved heme channel in Gram-negative bacteria [103]. In line with this finding, gain-of-function mutations in the β-barrel domain of heme receptor gene *hmuR* have allowed BD110 to utilize the xeno-siderophore desferrioxamine [40]. In SF45436Δ*fprA*-1, another adaptive mutation (W84G) was identified within the conserved NodB motif of b56940 that is a putative polysaccharide deacetylase (Fig. S7) belonging to the carbohydrate esterase 4 superfamily (CDD entry: cd10928). Enzymes of this family catalyze the removal of either N-linked or O-linked acetyl groups from *N*-acetylglucosamine or *O*-acetylxylose residues of their substrates including cell wall polysaccharides [104–106]. The W84G mutation in b56940 alone significantly enhanced the growth of SF45436Δ*fprA* under iron-limiting conditions [SF45436Δ*fprA* (b56940*); Fig. 6B] and, when combined with the c24120-T335-G336_del_ mutation, allowed a growth phenotype like the evolved clone SF45436Δ*fprA*-1 (Fig. 6B). SF45436Δ*fprA*::pB-SR6-1 showed a higher fitness than SF45436Δ*fprA*(c24120-D332-T333_del_) under the same iron-limiting conditions (Fig. 6C), indicating the contribution by both SR6 and this adaptive mutation. SR6 is functionally essential for ferrichrome intake in BD110 [40] and is a homolog of the characterized siderophore receptor FiuA that showed uptake activity for ferricrocin, ferrichrycin, and rhodotoluric acid in *P. aeruginosa* [81]. The result obtained in this work suggests that SR6 may contribute to the uptake of petrobactin in the presence of the observed adaptive mutations.

The adaptive mutations identified in the evolved clone SF45436Δ*fprA*::pB-SR5-1 include a frameshift mutation at L40 of ExbD (c12840). ExbD is a component of the TonB-ExbB-ExbD complex that delivers energy, generated by proton motive force, to support the outer membrane transport activity [82]. The transcription of *exbBD* is also regulated by the iron-responsive regulator RirA [33]. This frameshift mutation generates a truncated ExbD* of 44 amino acids lacking the periplasmic section while retaining the transmembrane α-helix and the key residue D25 (Fig. S7) involved in ExbB interaction and modulating conformational change of the TonB-ExbB-ExbD complex [107]. In addition to the stable ExbB-TonB interaction in the inner membrane, the periplasmic domain of ExbD can transiently interact with a periplasmic motif of TonB and is involved in switching the “close” and “open” states of the ExbB-ExbD complex regarding proton permeation [108]. Certain mutants in the periplasmic domain of ExbD can yield either open or closed state permeable to protons [108]. Reverse genetic evidence demonstrated that the frameshift mutation in ExbD of SF45436Δ*fprA* allowed partial restoration of growth under iron-limiting conditions (Fig. 6D). When this frameshift mutation of the evolved clone SF45436Δ*fprA*::pB-SR5-1 was complemented with the wild-type gene, bacterial fitness was partially reduced (Fig. 6D). These results indicate the contribution of adaptive mutation in the porin encoding gene c07550 and/or the introduced FepA homolog SR5 (Fig. 6A). FepA is a well-characterized siderophore receptor for transporting enterobactin, glucosylated enterobactin, and monocatecholate dihydroxybenzoic acid [31, 83]. The adaptive mutation (Y158C) of c07550 was identified within the β-barrel domain of porins (Fig. S6). Porins are trimeric β-barrel proteins integrated in the outer membrane, allowing passive diffusion of small solutes <600 Da. In contrast, petrobactin (C_34_H_50_N_6_O_11_) produced by SF45436 has a molecular weight of 718.8 Da and thus cannot permeate through porins. In *E. coli*, porins are important in iron intake when the high-affinity iron transport system, e.g. TonB-dependent siderophore pathway, is blocked [109]. Similarly, the porin of *Mycobacterium smegmatis*, a distinct Gram-positive bacterium possessing an outer membrane, has been demonstrated as a part of the low-affinity iron uptake system, and its mutation leads to overproduction of siderophores under iron-limiting conditions [110].

In the evolved clone SF45436Δ*fprA*::pB-SR1-1, the adaptive mutation (T91P) was associated with the ligand-binding pocket of the regulator subunit of cAMP-dependent protein kinase (b56780; PKAR1 homolog; Fig. S6) that is conserved in both prokaryotes and eukaryotes [111] (Fig. 6A). In *Mucor lusitanicus*, the *pkaR1* deletion mutation or a higher concentration of exogenous cAMP leads to reduced production of the siderophore rhizoferrin that is also found in bacteria [112, 113]. When the point mutation of the evolved clone SF45436Δ*fprA*::pB-SR1-1 was complemented with the wild-type *b56780*, the rescue effect was blocked (Fig. 6E; SF45436Δ*fprA*::pB-SR1-1-R). The T91P mutation of b56780 partially rescues the growth defect of SF45436Δ*fprA* under iron-limiting conditions [Fig. 6E; SF45436Δ*fprA*(b56780*)], indicating the contribution of the catecholate-group siderophore receptor SR1 (Fiu homolog) [83].

**Figure 7:**
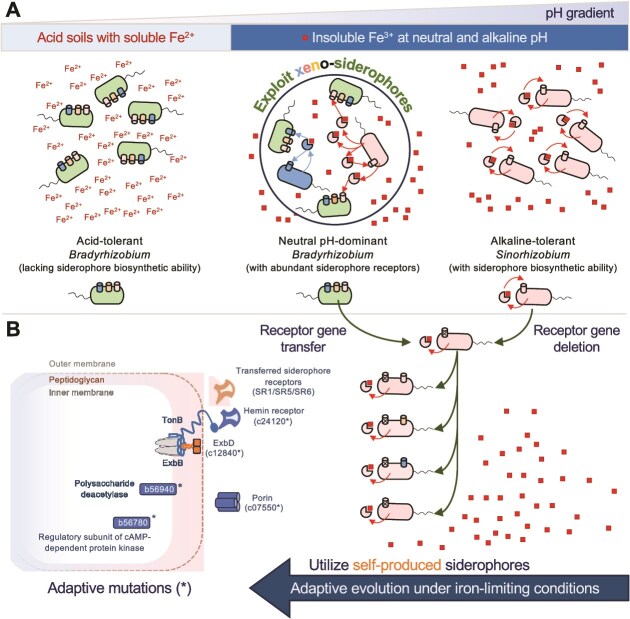
Evolutionary strategies of xeno- and self-produced siderophore utilization in soybean rhizobia. (A) Exploiting xeno-siderophores with more receptors. In nature, most *Bradyrhizobium* strains are acid-tolerant nonsiderophore producers but harbor more xeno-siderophore receptors of diverse groups than alkaline-tolerant siderophore-producing *Sinorhizobium*. Both natural (e.g. BD110 with eight xeno-siderophore receptors) and engineered (e.g. SF45436Δ*asbA* with two siderophore receptors) xeno-siderophore exploiters show a competitive advantage over siderophore-producing competitors (e.g. SF45436 with two receptors) and altruistic mutants (e.g. SF45436Δ*fprAfoxA*) under the tested iron-limiting conditions at neutral pH. (B) Rapid adaptive evolution of *Sinorhizobium* lacking the specific receptor compatible with the self-produced siderophore. The altruistic mutants (e.g. SF45436Δ*fprA* lacking the coevolved receptor for the self-produced petrobactin) or their derivatives carrying horizontally transferred siderophore receptors from *Bradyrhizobium* (e.g. SF45436Δ*fprA*::pB-SR1) show improved fitness under iron-limiting conditions in the experimental evolution study. Adaptive mutations are found in various functions (see Fig. 6 for more details).

## Conclusion

Most *Bradyrhizobium* and *Sinorhizobium* strains associated with soybeans grow well under acidic/neutral and alkaline/neutral conditions, respectively [4, 30]. *Sinorhizobium* strains dominate soybean nodules under alkaline conditions, whereas *Bradyrhizobium* strains are the major soybean microsymbionts under acidic and neutral conditions in both geographic landscape and common garden experiments [16, 29, 78, 93–96]. Evidently, the existing knowledge indicates that *Bradyrhizobium* prevails in the antagonistic interaction with *Sinorhizobium* under neutral conditions. This study further revealed that the ecological success of acid-tolerant *Bradyrhizobium* under neutral pH conditions can be at least partly attributed to its exploitation of xeno-siderophores from the competing *Sinorhizobium. Bradyrhizobium* members have evolved a distinct evolutionary trait of having abundant siderophore receptors of different groups but just occasionally possessing siderophore biosynthesis functions (Fig. S1 and Figs 1 and 2). Few acid-tolerant *Bradyrhizobium* members have siderophore biosynthesis ability, likely due to abundant soluble Fe^2+^ in acid soils. This evolutionary feature makes *Bradyrhizobium* strains canonical xeno-siderophore exploiters under neutral conditions (Figs 3 and 4) at the free-living stage (Fig. 3) and during competitive nodulation (Fig. 4). When siderophore receptor genes of *Bradyrhizobium* were transferred to a siderophore-producing *Sinorhizobium* mutant that lacks compatible receptors for the self-produced siderophore (Fig. 5), rapid adaptive evolution of machinery involved in utilizing self-produced siderophores can be achieved under iron-limiting conditions (Fig. 6). These evolutionary strategies are in line with the general biogeographic patterns of *Bradyrhizobium* and *Sinorhizobium* associated with soybeans. Most *Bradyrhizobium* strains living in iron-rich acid soils lack siderophore biosynthetic ability and recruit more siderophore receptors to exploit xeno-siderophores under iron-limiting neutral conditions (Fig. 7A). On the other hand, *Sinorhizobium* members produce their own siderophores to survive in iron-limiting alkaline or neutral conditions and have evolved a relatively flexible machinery for utilizing self-produced siderophores when the compatible siderophore receptor is missing (Fig. 7B). It is established that the level of soluble iron strongly depends on soil pH. Thus, the natural and experimental evolutionary insights into xeno- and self-produced siderophore utilization by soybean rhizobia (*Bradyrhizobium* and *Sinorhizobium*) can be generally important in shaping the biogeography of microorganisms. As soil pH varies considerably among different ecoregions and even between sites within the same field, a single inoculant of either *Bradyrhizobium* or *Sinorhizobium* cannot ensure an adequate number of rhizobial cells in the rhizosphere to support nodulation. This work provides eco-evolutionary mechanisms and theoretical bases for the development of a mixed inoculant containing both appropriate *Bradyrhizobium* and *Sinorhizobium* strains, enabling its application in different ecoregions with soil pH spanning acidic, neutral, and alkaline ranges.

## Supplementary Material

wraf280_Supplemental_Files

## Data Availability

All data generated or analyzed during this study are included in this published article [and its supplementary information files].
