# Supplementary material for: Evolution of rhizobial siderophore utilization via accessory xeno-siderophore receptors and flexible intake machinery for self-produced siderophores
Source: ISME J. 2025 Dec 19;20(1):wraf280. doi: 10.1093/ismejo/wraf280 (PMC12815270; doi:10.1093/ismejo/wraf280)
Supplement: wraf280_Supplemental_Files [file wraf280_supplemental_files.zip › Supplementary Figures S1-S7.pdf]

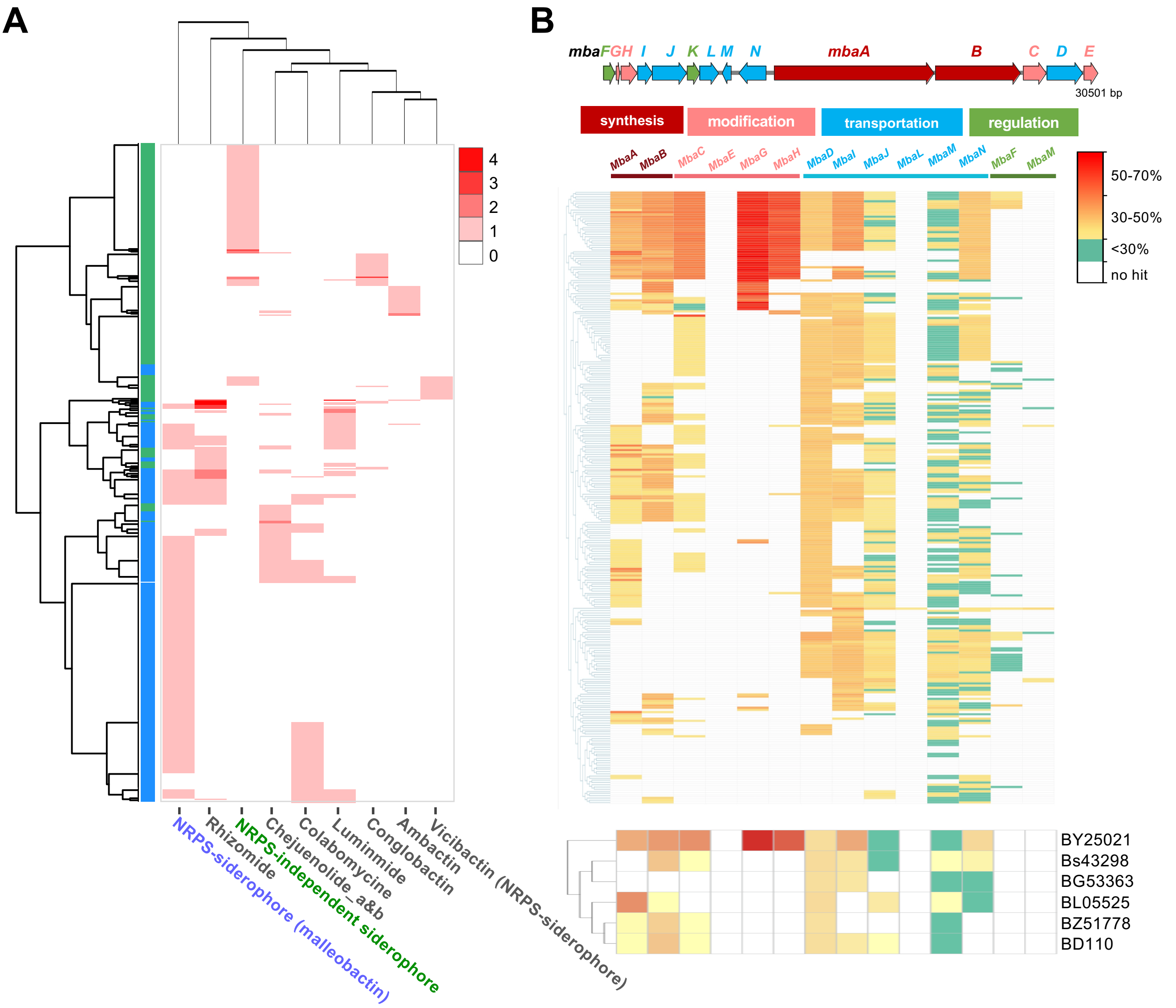

Fig. S1 Phyletic distribution of predicted secondary metabolism gene clusters in *Bradyrhizobium* and *Sinorhizobium*. (**A**) Hierarchical cluster analysis of phyletic distribution of top 5 gene clusters identified in 278 *Bradyrhizobium* (blue) or in 210 *Sinorhizobium* (green) genomes based on antiSMASH. (**B**) Phyletic distribution of the malleobactin biosynthetic proteins in *Bradyrhizobium*. The results for six *Bradyrhizobium* strains used in Fig 1 and Fig 2 are also shown at the bottom. BY, *Bradyrhizobium yuanmingense*; Bs, *Bradyrhizobium* sp.; BG, *Bradyrhizobium guangxiense*; BL, *Bradyrhizobium liaoningense*; BZ, *Bradyrhizobium zhanjiangense*; BD, *Bradyrhizobium diazoefficiens*. Heatmap shows protein identity values between Mba proteins directing malleobactin biosynthesis from *Burkholderia thailandensis* E264 and their corresponding homologs in each *Bradyrhizobium* strain. MbaE (formyltransferase) essential for obtaining functional malleobactin is absent in all 278 analyzed *Bradyrhizobium* strains.

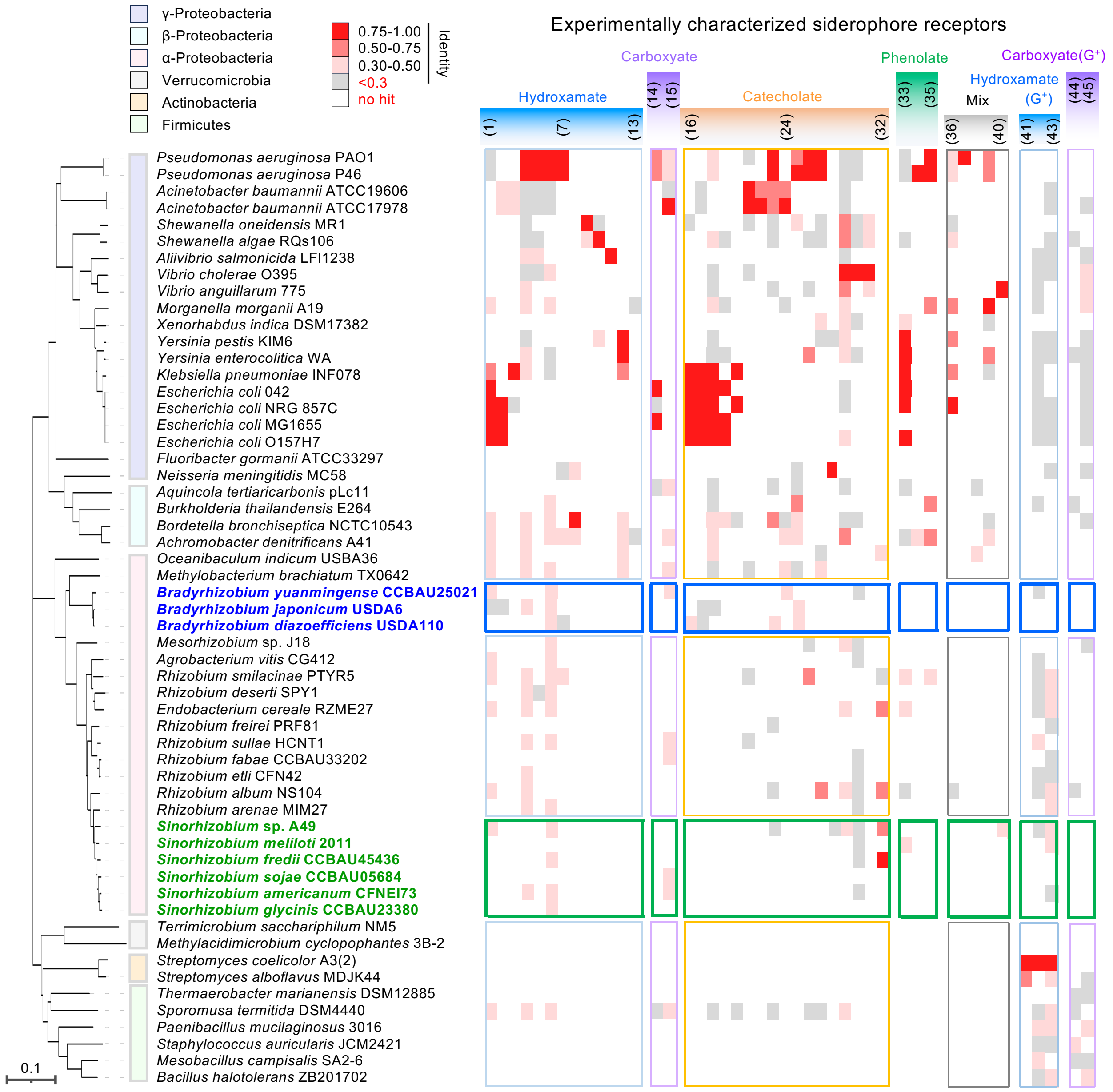

Fig. S2 Phyletic distribution of homologs of known siderophore receptors in representative bacteria. The neighbor-joining phylogenetic tree of representative species belonging to different phyla/classes was constructed based on RpoB. Gray color indicates that protein identity is less than 30%. Protein identity values are indicated by red scale of three grades. Reference siderophore receptors: (1) FhuA, (2) FhuE (3) FhuE2, (4) FoxA, (5) ChtA, (6) FiuA, (7) FpvB, (8) FauA, (9) PutA, (10) AvtA, (11) BitA, (12) FcuA, (13) RhtA, (14) FecA, (15) FbsN, (16) FepA, (17) Fiu, (18) CirA, (19) YddB, (20) IroN, (21) BfhH, (22) PiuA, (23) PirA, (24) BauA, (25) PiuD, (26) PfeA, (27) FvbA, (28) FrpB, (29) IrgA, (30) VctA, (31) ViuA, (32) FprA, (34) PupA, (35) FptA, (36) IutA, (37) FpvA, (38) PupB, (39) RumA, (40) FatA, (41) DesE, (42) CdtB, (43) CchF, (44) HtsA, (45) SirA. To avoid confusion regarding the nomenclature of receptors, the protein names reported in the original publications have been used, and relevant references have also been provided. Tree scale is 0.1.

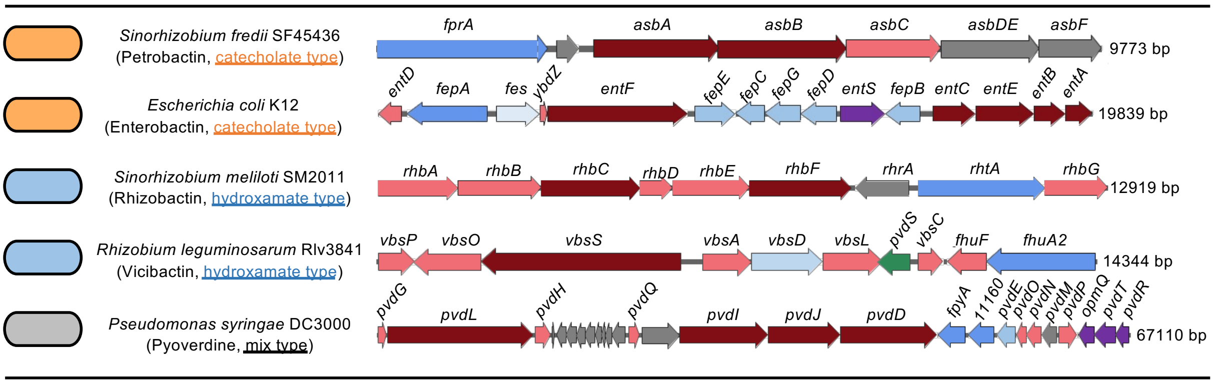

Fig. S3 Known siderophore categories and corresponding gene clusters in test siderophore producing bacteria. Siderophore types are indicated in orange (catecholate), blue (hydroxamate) and grey (mix type) colors. Gene clusters directing biosynthesis of corresponding siderophores are shown.

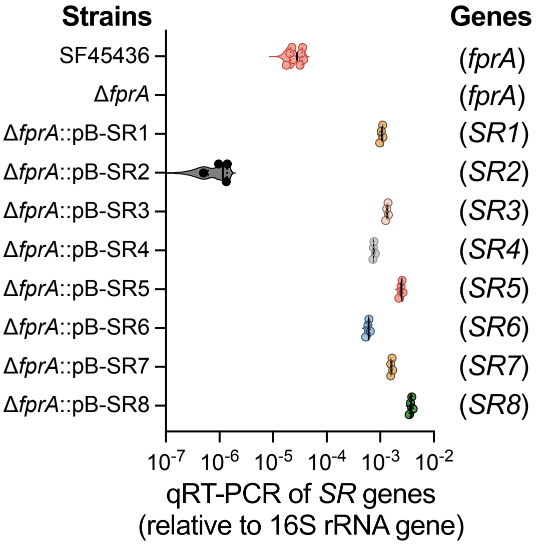

Fig. S4 qRT-PCR analyses of each siderophore receptor genes in SF45436 and its derivatives under iron-limiting conditions. Eight (SF45436) or four biological replicates (the other test strains) were tested.

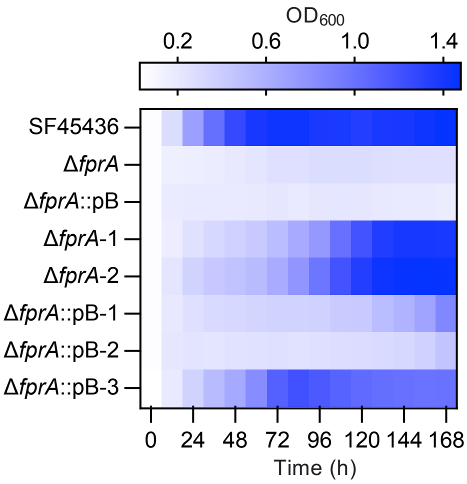

Fig. S5 Growth phenotypes of SF45436Δ*fprA* derivatives with or without the empty vector pBBR1MCS-5. Independent clones were collected at 128 hours post cultivation as mentioned in Fig 5, and further cultured under the same conditions in the experimental evolution scenario. The OD_{600} values were based on the mean of five biological replicates for all test strains. This experiment was performed independently from that shown in Fig 5. Evolved strains are labelled with “-1”, “-2”, and “-3”.

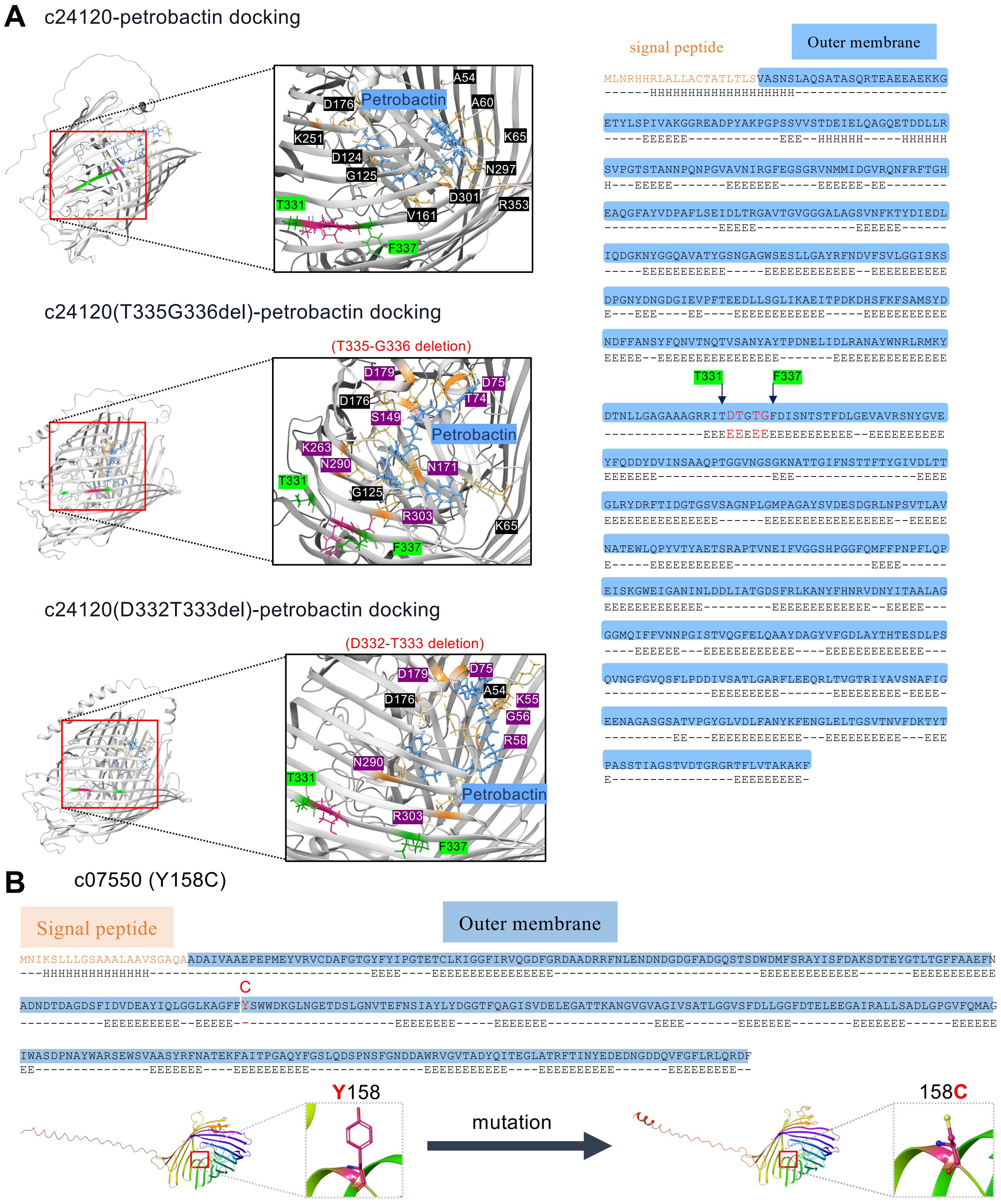

Fig. S6 Structure modeling analysis of outer membrane proteins harboring adaptive mutations. (**A**) c24120 and its derivatives harboring in-framing deletions of residues T335-G336 and D332-T333 within the β-barrel domain. Docking simulations between c24120 and petrobactin (CID: 11411510) was performed using Schrodinger Maestro 11.5. The key residues involved in petrobactin interactions are indicated on black and purple backgrounds. (**B**) c07550 and its derivative carrying the Y158C mutation within the β-barrel domain. The β-barrel domains of both c24120 and c07550 are integrated into the outer membrane. Secondary and 3D structures were predicted by JPred4 and AlphaFold 3.0, respectively. Subcellular localization was predicted using PSORTb, CELLO v.2.5, and DeepLoc 2.0. The docking between c24120 and petrobactin (CID: 11411510) was performed using Schrodinger Maestro 11.5.

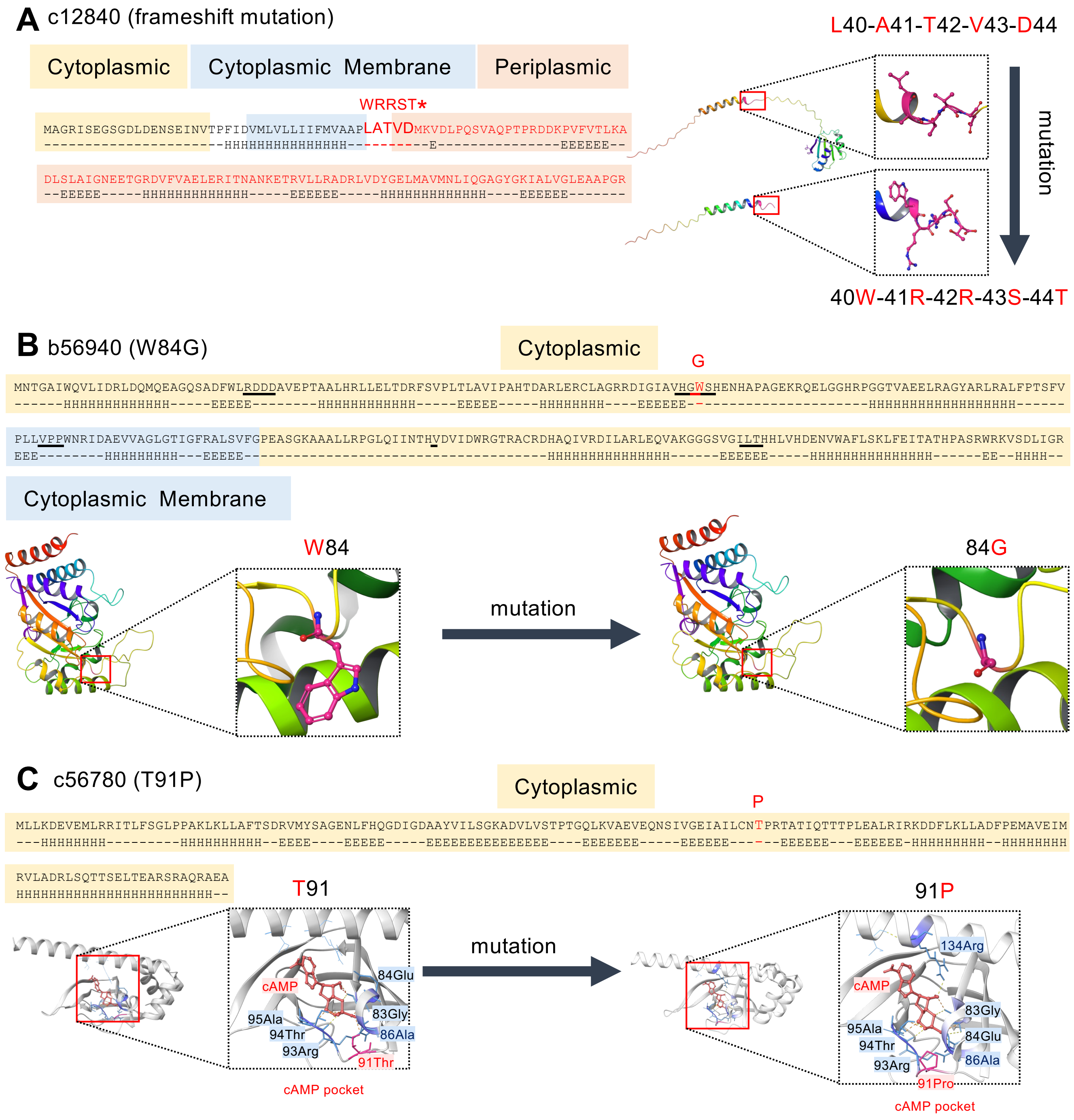

Fig. S7 Structure modeling analysis of cytoplasmic or inner membrane proteins harboring adaptive mutations. (**A**) c12840 and its derivative harboring a frameshift mutation starting at L40. *, indicates premature termination caused by a nonsense mutation. (**B**) b56940 and its derivative carrying W84G mutation within the conserved NodB motif (underlined residues) of polysaccharide deacetylase belonging to the carbohydrate esterase 4 superfamily (CDD entry: cd10928). (**C**) c56780 and its derivative harboring the T91P mutation within the cAMP binding pocket of regulator subunit of cAMP-dependent protein kinase. Secondary and 3D structures were predicted by JPred4 and AlphaFold 3.0, respectively. Subcellular localization was predicted using PSORTb, CELLO v.2.5, and DeepLoc 2.0. Docking simulations between c56780 and cAMP (CID: 6076) was performed using AutoDock Vina method via SwissDock.
